# Precursor-derived in-water peracetic acid impacts on broiler performance, gut microbiota, and antimicrobial resistance genes

**DOI:** 10.1016/j.psj.2022.102368

**Published:** 2022-12-01

**Authors:** Salvatore Galgano, Leah Conway, Francesco Di Maggio, Kathryn Farthing, Nikki Dalby, Adrian Fellows, Jos G.M. Houdijk

**Affiliations:** ⁎ Monogastric Science Research Centre, Scotland's Rural College, Edinburgh, Scotland, United Kingdom; † Gama Healthcare Ltd and Aga Nanotech Ltd, Halifax, United Kingdom; ‡ Centre for Innovation Excellence in Livestock, York, United Kingdom

**Keywords:** peracetic acid, microbiota, antimicrobial resistance, antimicrobial alternative, broiler

## Abstract

Past antimicrobial misuse has led to the spread of antimicrobial resistance amongst pathogens, reportedly a major public health threat. Attempts to reduce the spread of antimicrobial resistant (**AMR**) bacteria are in place worldwide, among which finding alternatives to antimicrobials have a pivotal role. Such molecules could be used as “green alternatives” to reduce the bacterial load either by targeting specific bacterial groups or more generically, functioning as biocides when delivered in vivo. In this study, the effect of in-water peracetic acid as a broad-spectrum antibiotic alternative for broilers was assessed via hydrolysis of precursors sodium percarbonate and tetraacetylethylenediamine. Six equidistant peracetic acid levels were tested from 0 to 50 ppm using four pens per treatment and 4 birds per pen (i.e., 16 birds per treatment and 96 in total). Peracetic acid was administered daily from d 7 to 14 of age whilst measuring performance parameters and end-point bacterial concentration (**qPCR**) in crop, jejunum, and ceca, as well as crop 16S sequencing. PAA treatment, especially at 20, 30, and 40 ppm, increased body weight at d 14, and feed intake during PAA exposure compared to control (*P* < 0.05). PAA decreased bacterial concentration in the crop only (*P* < 0.05), which was correlated to better performance (*P* < 0.05). Although no differences in alpha- and beta-diversity were found, it was observed a reduction of *Lactobacillus* (*P* < 0.05) and *Flectobacillus* (*P* < 0.05) in most treatments compared to control, together with an increased abundance of predicted 4-aminobutanoate degradation (V) pathway. The analysis of the AMR genes did not point towards any systematic differences in gene abundance due to treatment administration. This, together with the rest of our observations could indicate that proximal gut microbiota modulation could result in performance amelioration. Thus, peracetic acid may be a valid antimicrobial alternative that could also positively affect performance.

## INTRODUCTION

Chicken gut microbiota consists of a multitude of microbial symbionts longitudinally colonizing the gastrointestinal tract, whose interactions with the host affect well-being and performance at several levels, including nutritional, immunological, and physiological (Diaz Carrasco et al., 2019). Host colonization is thought to start soon after hatching, with low parental contribution, and presents both temporal and longitudinal fluctuations, which primarily depend on environmental factors (Stanley et al., 2014). Due to the well-documented interactions between microbiota and host, manipulation of the microbial communities through probiotics (Memon et al., 2021), prebiotics (Ricke, 2021), and for several decades also via antimicrobial growth promoters (**AGP**) (Collignon, 2004; Costa et al., 2017) has been applied to impact broiler production. However, past AGP misuse has led to an increased antimicrobial resistance (**AMR**) among pathogens, characterized as a major health threat for both animals and humans alike (Munita and Arias, 2016).

The issue of AMR associated with poultry production has reached concerning levels (Nhung et al., 2017; Agyare et al.), thus alternative to antibiotics, such as for example probiotics, prebiotics, organic acids, and plant extracts have been proposed (Lewith and Jacob, 2005). Other type of alternatives may also exert antimicrobial activity, include enzymes, organic acids, immunostimulants, bacteriocins, bacteriophages, phytogenic feed additives, phytoncides, nanoparticles, and essential oils (Mehdi et al., 2018). Peracetic acid (**PAA**) is an emerging biocide widely used in contexts beyond poultry production, for example, wastewater treatment (Zhang et al., 2020), or poultry processing with proven inhibitory effect on pathogens such as *Campylobacter* (Micciche et al., 2019). Here, we propose and test the possible in vivo effect of PAA in broiler birds administered through water, on gastrointestinal microbial communities, main AMR gene relative abundance and performance. The generic antimicrobial activity of PAA action We tested the broad-spectrum antimicrobial activity of different PAA levels of inclusion at the end of the starter phase, for 7 d, on young birds during a 14-d trial without interfering with the normal microbiota colonization dynamics through the first week, hence the choice of administering PAA only from d 7 to d 14. We observed positive effects on performance, correlated to a reduction of bacterial concentration in the crop and specifically to a significant reduction of *Firmicutes* at phylum level and *Lactobacillus* at genus level. Our findings indicate both the possible use of in-water PAA as an antimicrobial alternative and the importance of the upper gut microbiota in broiler performance in young broiler birds.

## MATERIALS AND METHODS

### Animal Study

The animal study was carried out at the Allermuir Avian Innovation and Skills Centre (**AISC**), SRUC. Study design and protocol were approved by SRUC Animal Welfare and Review Body (POU AE 20-2019). A total of 96-day-old Ross 308 male broilers were placed in 24 pens within 2 rooms, with 4 birds per pen and 4 pens per treatment (stocking density at d 14: ∼1.9 Kg/m^2^), with a total of 6 treatments (Table 1) between d 7 and d 14 of age, which was the last day of the trial. Chickens were fed standard commercial wheat, soybean meal based diet formulated to have 20.8% crude protein, 1.21% d-lysine, and 12.8% apparent metabolizable energy. Feed was offered as starter diet (mash, ad libitum) throughout the study designed to meet standard nutrient requirements (crude protein: ∼23%, 2,800 Kcal metabolizable energy/Kg). At d 0 all the birds were wing-tagged and allocated to one of the 24 pens following a randomized complete block design, where treatments were also randomly allocated within each of 4 blocks. Number of birds and replicate was based on a number of previous dose-response studies with a similar design, in which 4 replicates and a set of orthogonal contrast statements were used to assess linear and quadratic effects of equidistant treatment levels (Smith et al., 2013).

**Table 1:** Water treatments from day 7 to day 14 of age. The different PAA levels of inclusion were obtained by mixing different concentrations of SP and TAED and by adding EDTA and citric acid as stabilizers. Control at 0 ppm was obtained by adding EDTA only.

Treatment	PAA level of inclusion	TAED (g/l)	SP (g/l)	NaEDTA (g/l)	Citric acid (g/l)
1	0 ppm	0	0	0.05	0
2	10 ppm	0.035	0.065	0.05	0.04
3	20 ppm	0.055	0.095	0.05	0.05
4	30 ppm	0.07	0.13	0.05	0.07
5	40 ppm	0.09	0.17	0.05	0.09
6	50 ppm	0.10	0.20	0.05	0.1

Abbreviations: NaEDTA, ethylenedinitrilotetraacetic acid disodium salt; PAA, peracetic acid; SP, sodium percarbonate; TAED, tetraacetylethylenediamine.

### Treatment Preparation

Peracetic acid (**PAA**) was produced in water by hydrolysis of precursors sodium percarbonate (**SP**) and tetraacetylethylenediamine (**TAED**) (AGA Nanotech, Hemel Hempstead, UK). In addition, disodium ethylenediaminetetraacetic acid (**EDTA**) was used as a stabilizer to prevent PAA degradation and citric acid was added to counterbalance the effect of SP and TAED on pH (Table 1). Water treatments were freshly prepared and administered to the chicken daily. The control at 0 ppm of PAA was prepared by addition of EDTA only as vehicle control. The precise precursor ratio needed to obtain the different PAA levels of inclusion (Table 1) were established in vitro prior to the animal study via measuring the PAA concentrations at each level using the using the free and total chlorine AccuVac (HACH, Loveland, CO) method as described below. Permachem N,N-diethyl-p-phenylenediamine total Chlorine reagent (HACH, product number 2105628) was added to 10 mL of TAED, SP, EDTA, and citric acid solution. Therefore, the absorbance at 530 nm was read via using the HACH spectrophotometer (DR6000 UV-VIS, HACH). Thus, PAA concentration was calculated from Chlorine values via multiplying Cl_2_ output of the spectrophotometer reads by 1.07 (i.e., scaling factor between chlorine and PAA concentration) and the dilution factor.

### Performance Analysis

Individual body weight (**BW**), feed issued, and feed refusals were measured at d 0, d 7, d 10, and d 14 to allow measurement of bird-level BW, bird-level body weight gain (**BWG**), pen-level feed intake (**FI**), and pen-level feed conversion ratio (**FCR**). Mortality correction for the latter was not required as there was no mortality before or during the experimental phases.

### Sampling

At d 14, all the birds were humanely culled via cervical dislocation, and content from crop, jejunum, ceca, and colon were pooled by pen and gut segment. pH was measured in the colon content (1111105 2-star benchtop pH meter, Thermo Scientific, Waltham, MA) while the content of the other segments was snap frozen at ∼−78°C (dry ice) before being transferred to an ultra-low-temperature freezer (−80°C) pending further analysis.

### DNA Isolation

Approximately 0.25 g of gut content were transferred in the PowerBead tubes of the DNeasy PowerSoil Kit while being mixed with 60 µL of solution C1 of the same kit (Part no. 12888-100, QIAGEN, Hilden, Germany). The tubes were placed in a FastPrep-24TM 5G homogenizer (116005500, MP Biomedicals, Irvine, CA) for 55 s at 5.5 m/s. Afterward, QIAGEN 12888-100 manufacturer instructions were followed to isolate total DNA, which was immediately stored at −80°C until further analysis.

### Bacterial Absolute Quantification

#### Standard Curve Preparation

Standard curve for absolute qPCR quantification was built through nine 10-fold serial dilutions of linear plasmid (Hou et al., 2010) containing qPCR target as insert. The latter was amplified from the isolated DNA through PCR reaction and separated via 1.5% agarose gel after electrophoresis at 100V for 80 min. The 25 µL-reaction mix included 1X of KAPA Taq ReadyMix with dye (Kapa Biosystems, Wilmington, DE), 0.2 µM of each primer (Table 2) and nuclease-free water. PCR conditions were 95°C for 3 min, 35 cycles including 95°C, 60°C both for 30 s and 72°C for 1 min, followed by a final elongation at 72°C for 10 min.

**Table 2:** List of primers used in this study to amplify the AMR genes and the V3 region of the 16S rRNA gene.

Resistance/target (class, gene)	Primers (5’ → 3’)	Annealing	Amplicon	Reference
Streptomycin, spectinomycin (Aminoglycoside, *aadA*)	Fw: GCAGCGCAATGACATTCTTG Rev: ATCCTTCGGCGCGATTTTG	60°C	282 bp	(Esperón et al., 2018)
Vancomycin (glycopeptide, *vanA*)	Fw: GCCGGAAAAAGGCTCTGAA Rev: TTTTTTGCCGTTTCCTGTATCC	60°C	90 bp	(He et al., 2020)
Vancomycin (glycopeptide, *vanC*)	Fw: CTTATGTTGGTTGCCATGTCG Rev: CGATTGTGGCAGGATCGTT	60°C	138 bp	(Flipse et al., 2019)
Tetracycline (*tetW_)_*	Fw: AGCGACAGCGTGAGGTTAAA Rev: AAGTTGCGTAAGAGCGTCCA	60°C	153 bp	(Juricova et al., 2021)
Tetracycline (*tetQ*)	Fw: AGAATCTGCTGTTTGCCAGTG Rev: CGGAGTGTCAATGATATTGCA	63°C	167 bp	(Aminov et al., 2001)
Methicillin, penicillin (β-lactam, *mecA*)	Fw: AACCACCCAATTTGTCTGCC Rev: TGATGGTATGCAACAAGTCGTAAA	60°C	135 bp	(Kelley et al., 2013)
V3 region 16S rDNA	341F: CCTACGGGAGGCAGCAG 518R: ATTACCGCGGCTGCTGG	60°C	192 bp	(Muyzer et al., 1993)

The amplicons were excised from gel, purified following the protocol from Wizard SV Gel and PCR Clean-Up System (Promega, Madison, WI) and cloned into a pCR2.1 plasmid vector (TA CloningTM Kit, Thermo Fisher Scientific, Waltham, MA), prior to transformation of ligase reaction into chemically competent One shot INVαF’ *E. coli* cells (Thermo Fisher Scientific) by heat shock.

The plasmid was isolated from liquid Luria-Bertani cultures inoculated with positive-X-gal-transformed colonies through the QIAprep Miniprep kit as per manufacturer instructions (27104, QIAGEN,). Insert presence was verified both by EcoRI (R3101S, New England BioLabs, Ipswich, MA) restriction enzyme digestion and by Sanger sequencing (DNA Sequencing and Services, Medical Sciences Institute, School of Life Sciences, University of Dundee). Finally, plasmids were linearized using 5 units of HindIII (R3104S, New England BioLabs) and 1X of CutSmart buffer (B7204, New England BioLabs) in 50 μL total volume.

Linear plasmid-copy number (**CN**) concentration was calculated from NanoDrop spectrophotometer ng/µL reads (TM 1000, Thermo Fisher Scientific) and further used through the qPCR reactions.

#### Absolute qPCR Quantification

Absolute qPCR quantification was carried to quantify total number of bacteria by targeting the V3 region of the 16S rRNA gene (Table 2). All reactions were carried out in 20 µL containing 1X of Takyon qPCR MasterMix with Low Rox (UF-LSMT-B0701, Eurogentec, Seraing, Belgium), 50 nM of each primer (Table 2), 10 ng of DNA template and nuclease-free water (129114, QIAGEN). Cycling conditions (Mx3000P thermocycler, Agilent Technologies, Santa Clara, CA) were 95°C for 3 min followed by 40 cycles at 95°C for 5 s and 65°C for 35 s, at the end of which fluorescence was detected. Qualitative template control was performed through melting curve analysis.

All the reactions were run in triplicate. Excellent reaction efficiency metrics were detected throughout the analysis, based on R^2^, slope and efficiency of the standard curve, whose average values were calculated as ∼0.99, ∼−3.3, and ∼100%, respectively.

#### qPCR Data Analysis

Copy number (CN) per reaction for each sample was calculated based on the linear regression model fitted with standard curve fluorescence and cycle threshold (Stratagene Mx3000P software, Agilent technologies).

Therefore, CN per reaction was first converted into bacterial cells per reaction (BC_r_) by normalizing CN to 5.2 average copy number of 16S gene per bacterial cell at the time of writing (Stoddard et al., 2015). Finally, BC per gram of sample was calculated using equation (1) below (Singh et al., 2014).

(1) $$\frac{BC_{r}\cdot C\cdot D}{S\cdot V}.$$

Where, C and D were concentration and dilution volume of the extracted DNA, respectively, while S was the amount of DNA subjected to qPCR and V was the amount of sample used to isolate DNA (Singh et al., 2014).

### Antimicrobial Resistance Gene Analysis

Table 2 depicts primers and annealing conditions applied through the relative qPCR quantification of the 6 AMR poultry-relevant genes selected. Each reaction (20 µL) was run in triplicate and included 1X Brilliant III Ultra-Fast SYBR Green qPCR Master Mix (600882, Agilent technologies), 1ng of crop content gDNA template and nuclease-free water (129114, QIAGEN), 250 nM of each primer for *tetW, vanC, aadA*, and 350 nM for *vanA, tetQ*, and *mecA*. Each array included a non-template control and the samples from the control group to eliminate inter-run bias while melting curve analysis assessed reaction quality. Amplification conditions (Mx3000P thermocycler, Agilent Technologies) were 95°C for 3 min followed by 40 cycles of 95°C for 10 s and 20 s annealing as per Table 2.

Fold-change relative abundance (i.e., 2^−ΔΔCt^) as per protocol used by other authors (Walsh et al., 2011; Juricova et al., 2021) was calculated as the Ct difference between the AMR genes and the 16S rRNA as a normalizer (ΔCt) between treatments and controls (ΔΔCt).

### 16S rRNA Gene Sequencing

#### Library Preparation

16S rRNA gene sequencing on crop-content gDNA was carried out by Omega Bioservices (Norcross, GA) targeting the V4 region of the bacterial 16S rRNA gene (F515_b_ (Parada et al., 2016): 5′-TCGTCGGCAGCGTCAGATGTGTATAAGAGACAGGTGYCAGCMGCCGCGGTAA-3’; R806_b_ (Apprill et al., 2015): 5′-GTCTCGTGGGCTCGGAGATGTGTATAAGAGACAGGGACTACNVGGGTWTCTAA-3’).

Amplicon PCR (total volume of 25µL) components (final concentration) were, 12.5 ng of template DNA,1x KAPA HiFi HotStart ReadyMix (KK3604, Kapa Biosystems) and 0.2 µM of each primer, whereas amplification conditions were 95°C for 3 min (initial denaturation) followed by 25 cycles of denaturation (95°C, 30 s), annealing (55°C, 30 s) and extension (72°C, 30 s), and a final elongation of 5 min at 72°C. PCR product clean-up was carried out using Mag-Bind RxnPure Plus magnetic beads (M1378-01, Omega Bio-tek). A second index PCR amplification, used to incorporate barcodes and sequencing adapters was performed maintaining the component concentrations as described above. Cycling conditions were 95°C for 3 min, followed by 8 cycles of 95°C, 55°C, and 72°C, each held for 30 s, thus a final 5-min elongation step at 72°C. Finally, the libraries ∼600 bases in size were checked using a 2200 TapeStation (5067, Agilent technologies) and quantified using QuantiFluor dsDNA System (E2671, Promega) before normalization, pooling, and sequencing (2 × 300 bp paired end read setting) on the MiSeq (SY-410-1003, Illumina, San Diego, CA).

#### Bioinformatic Analysis

A total of 5,480,595 FASTQ paired end demultiplexed reads (∼ 230,000 × 2 reads/sample) were imported and analyzed in QIIME2 v2022.2 (Bolyen et al., 2019), through which ∼105,000 reads/sample were retained after being joined via VSEARCH (Rognes et al., 2016), with quality score of ∼40 throughout the sequence length both before and after quality-filtering with minimum Phred score of 20 (McKinney, 2010; Bokulich et al., 2013). Therefore, Deblur was used to denoise, with sequence trimming set at 290 bp (Amir et al., 2017) and taxonomy was assigned using the q2-feature-classifier plugin via applying a Naïve Bayes classifier, trained based on the F515_b_/R806_b_ primers and the last release of the Silva data base (138, 99% of similarities) (Pruesse et al., 2007; Pedregosa et al., 2011; Bokulich et al., 2018). Diversity analysis was carried out on even sequence depth of 3,790, retaining 90,960 (48.61%) features in 24 (100%) samples, allowing calculation of α-diversity through richness and Shannon's diversity index (Anderson, 2001; Kim et al., 2017), testing for significance through the Kruskal-Wallis test (Kruskal and Wallis, 1952; Benjamini and Hochberg, 1995). Moreover, β-diversity was measured through the Bray-Curtis dissimilarities and the Jaccard similarity index (Jaccard, 1908; Bray and Curtis, 1957), followed by permutational multivariate analysis of variance (**PERMANOVA**) (Anderson, 2001). Functional gene prediction based on 16S data was carried out via using PICRUSt2 (Langille et al., 2013). Differential abundance analysis was carried out via Microbiome Multivariable Association with Linear Models 2 (MaAsLin2, Mallick et al., 2021) in R v4.1.2 (R Core Team, 2021). This included the use of a negative binomial distribution (NEGBIN, Zhang et al., 2017) for differential microbial abundance analysis on cumulative sum scaling (**CSS**) normalized taxonomical data in order to reduce the bias introduced by differences in sampling depth (Pereira et al., 2018). NEGBIN was also used on trimmed mean of M values (**TMM**) for metabolic pathways prediction output of PICRUSt2 to account for the homological content of transcriptomic data (Robinson and Oshlack, 2010).

The package qiime2R (Bisanz, 2018) was used to graphically represent QIIME2 outputs produced through the analysis.

### Statistical Analysis

Linear mixed model (**LMM**) was carried out to assess whether treatments had a significant impact on the variables analyzed, fit with linear and quadratic treatment terms allowing the assessment of eventual dose response effect. All the analyses were carried out in R (R Core Team, 2021), were the LMM was fitted using “*lmer*” function from the *lme4* package (Bates et al., 2015), thus “*lmerTest*” was applied to calculate the *P* value for the *t* tests output of *lme4* through using Satterthwaite's method (Kuznetsova et al., 2017). In addition, both linear and quadratic terms were incorporated with a contrast analysis within “*lmer*” allowing the identification of a possible optimum level. Metabolic pathways predicted via PICRUSt2 pipeline were analyzed through alpha- and beta-diversity via the Vegan package in R and differential abundance analysis was carried out through NEGBIN on TMM normalized data via Maaslin2.

Treatment and time, for longitudinal data, were both input as fixed effects in LMM, whereas the hierarchy of “Room/Block/Pen/Bird” represented the random effects, the model was fitted with random intercept. Finally, Tukey corrected post hoc comparison for significant regressions or interactions, was carried out through using the function “*emmeans*” (Lenth, 2021). Longitudinal BW was Log_10_ transformed prior to fitting the model.

## RESULTS

### Performance and Colonic pH

Results from the analysis of the different performance parameters and colonic pH are summarized in Table 3. As expected, there was a significant effect of time to the cumulative BW at bird-level (F(2,180) = 8097.01, *P* < 0.01). At d 14, birds on the 20, 30, and 40 ppm PAA treatments were 10.6, 10.0, and 12.5% heavier (*P* < 0.05) than the control birds. This effect was less pronounced for birds allocated to the 10 and 50 ppm PAA treatments, which were 7.3% (*P* = 0.14) and 6.1% (*P* = 0.21) heavier than the control birds, respectively). This concurred with a significant quadratic effect of PAA inclusion on both longitudinal BW (F(2,92) = 4.5, *P* = 0.01) and d 14 BW (F(2,92) = 4.10, *P* = 0.02).

**Table 3:** Performance and colonic pH data at different intervals through the treatment week. Means ± standard deviations are shown for each treatment according to the detailed number of observations.

	Average body weight/period (Kg, d 14)	Average feed intake/bird/period (Kg, d 7–14)	Average FCR/bird/period (d 7–14)	Colonic pH (d 14)
0 ppm	0.432 ± 0.065^A^	1.37 ± 0.1^a^	1.23 ± 0.06	6.94 ± 0.55
10 ppm	0.464 ± 0.067	1.45 ± 0.08	1.24 ± 0.08	7.32 ± 0.58
20 ppm	0.478 ± 0.061^B^	1.53 ± 0.05^b^	1.24 ± 0.01	6.94 ± 0.40
30 ppm	0.475 ± 0.056^B^	1.51 ± 0.11^b^	1.30 ± 0.14	6.88 ± 0.88
40 ppm	0.486 ± 0.049^B^	1.51 ± 0.04^b^	1.26 ± 0.04	7.17 ± 0.24
50 ppm	0.459 ± 0.066	1.43 ± 0.13	1.25 ± 0.05	7.34 ± 0.43

ab;AB Different superscripts in the same row indicate statistically significant differences (*P* < 0.05 and *P* < 0.10 if upper or lowercase, respectively) output of the linear mixed model and Tukey corrected post hoc comparison of the type III LMM analysis of variance with Satterthwaite's method. BW (n = 16), FI, FCR and pH (n = 4).

Pen level FI analysis revealed increased FI from d 7 to d 14 (F(5,17) = 2.17, *P* = 0.107), with a clear positive effect birds on the 20, 30, and 40 ppm PAA treatments, recording 0.15, 0.13, and 0.13 Kg more, respectively compared to the 1.37 kg for the control birds (*P* < 0.05). Pen level FCR did not differ between PAA treatments, while colonic pH did not change among the experimental conditions.

### Total Bacterial Quantification and Correlation With Performance

**Figure 1:**
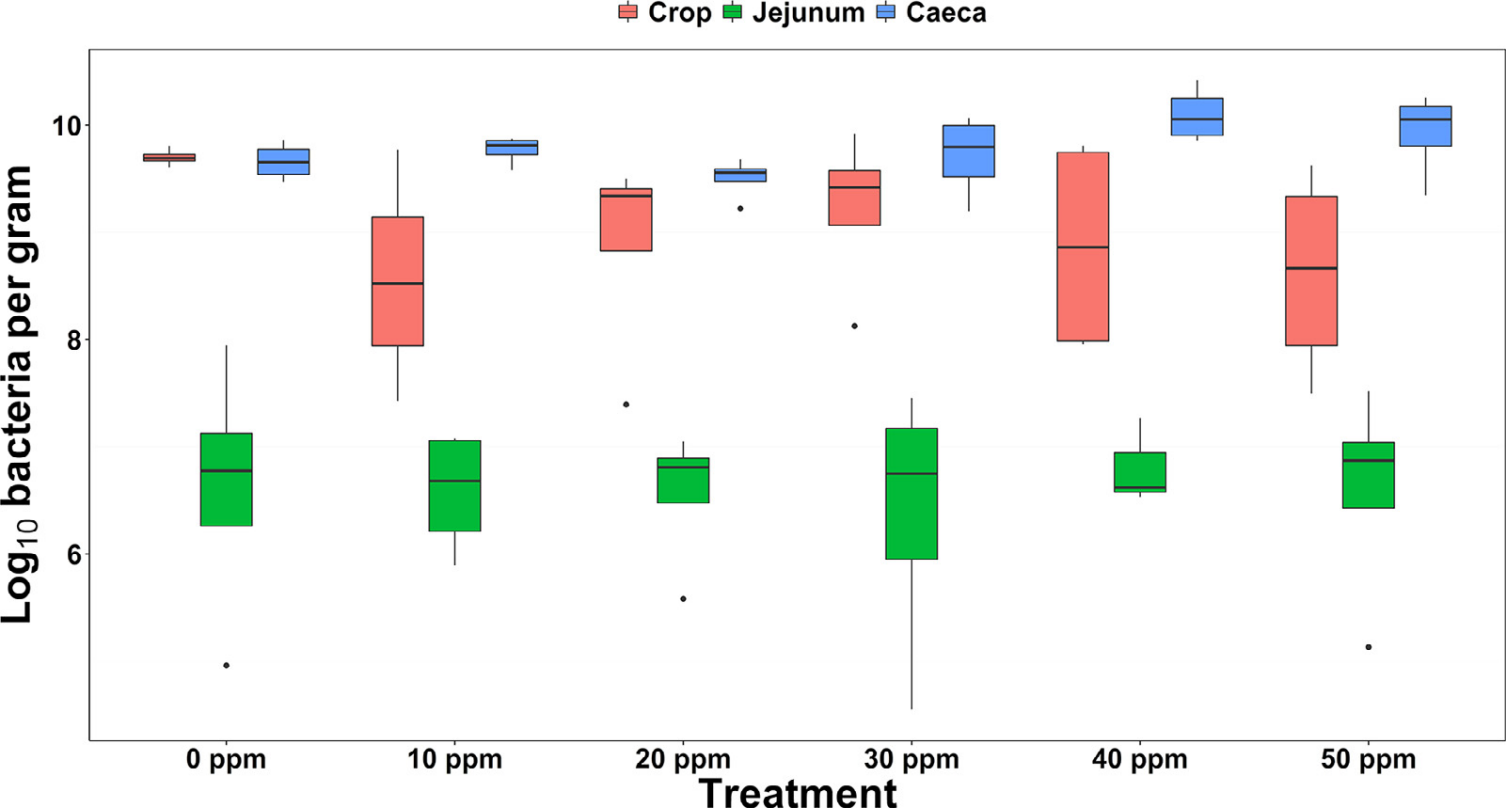
Log10 bacterial concentration in the three different gut locations analyzed.

As expected, bacterial concentration differed between crop, jejunum, and cecal content (F(2,35.9) = 133.57, *P* < 0.01, Figure 1). However, PAA treatment impacted on bacterial abundance in the crop only. Bacterial concentration (mean log_10_±SD) was reduced at PAA levels of 10 ppm (8.56 ± 1.02; *P* < 0.05), 20 ppm (8.89 ± 1.00; *P* = 0.103), 40 ppm (8.87 ± 1.04; *P* = 0.09), and 50 ppm (8.61 ± 0.99; *P* < 0.05) compared to the control (9.70 ± 0.08). Bacterial concentration in the jejunum and cecal content did not differ between PAA treatments and averaged 6.59 ± 0.87 and 9.78 ± 0.26, respectively, though cecal bacterial concentration appeared greatest for the 40 ppm and 50 ppm treatments.

**Figure 2:**
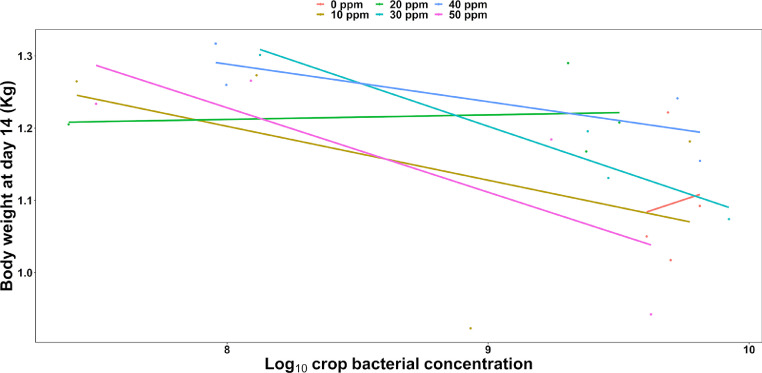
Correlation between body weight gain and Log10 crop bacterial concentration. Each dot represents the bacterial abundance associated with the different treatments (in different colors), whereas the size of the dot indicates whether the measurement was associated with either of the rooms. Linear models for each treatment are represented by the colored lines, indicating the monotonic inverse relationship between the variables especially for treatments whose level of inclusion was higher than 20 ppm.

To explore possible relationships between bacterial load and performance, BW at d 14 was fitted in an additional LMM as a depended variable and crop bacterial concentration as a fixed effect, again with room/block representing the hierarchy of random effects, this revealed an inverse relationship between the 2 variables (F(1,22) = 8.47, *P* < 0.01, Figure 2), with better performance (greater BW at d 14) for birds with lower crop bacterial concentration. Indeed, the model demonstrated that high bacterial concentrations as observed in the control birds concurred with reduced performance at endpoint, compared to birds on PAA treatments, which was associated with greater final BW and lower crop bacterial abundance.

### Crop Antimicrobial Resistance Gene Analysis

Six AMR genes were analyzed in total by relative quantification using the ΔΔCt method using the 16S rRNA gene as a normalizer and read-outs for the control birds as baseline (Table 4). While there were no significant linear regressions between PAA level and any AMR gene relative abundance, a quadratic relationship was observed between PAA and tetQ relative abundance (F(2,21) = 4.05, *P* = 0.03), arising from a decrease associated with 10, 20, and 30 ppm PAA compared to the control, followed by an increased gene abundance at 40 and 50 ppm PAA. In addition, a tentative quadratic relationship was observed between PAA and mecA (F(2,18) = 2.72, *P* = 0.09), arising from an increased gene level at 20, 30, and 40 ppm PAA compared to control and 50 ppm PAA. Furthermore, although AMR relative abundance was associated with rather large variance throughout, compared to the control, relative abundance was greater for aadA at 50 ppm PAA, vanC at 40 ppm PAA, and mecA at 20 ppm PAA (all *P* < 0.05) and mecA at 40 ppm PAA at *P* = 0.09.

**Table 4:** Mean fold change relative abundance (±standard deviation) of the six AMR genes analyzed through relative qPCR with the 2^−ΔΔCt^ method, using the 16S rRNA gene as normalizer and the 0ppm control for comparison.

PAA	aadA	*P* value	tetQ	*P* value	vanC	*P* value
0 ppm	1.36 ± 0.93^A^		1.74 ± 1.48		0.75 ± 0.22^A^	
10 ppm	4.6 ± 4.88	0.12	0.12 ± 0.03	0.43	0.72 ± 0.1	0.99
20 ppm	3.86 ± 2.73	0.23	0.35 ± 0.21	0.50	0.86 ± 0.07	0.95
30 ppm	3.21 ± 2.96	0.37	0.32 ± 0.35	0.49	1.36 ± 1.36	0.76
40 ppm	2.76 ± 2.81	0.50	2.04 ± 1.94	0.89	5.15 ± 5.57^B^	0.03
50 ppm	8.88 ± 9.04^B^	<0.01	2.99 ± 3.84	0.54	1.48 ± 0.97	0.72

PAA	tetW	*P* value	vanA	*P* value	mecA	*P* value

0 ppm	2.19 ± 2.74		1.54 ± 1.2		1.13 ± 0.58A,a	
10 ppm	1.89 ± 1.03	0.43	0.48 ± 0.21	0.61	1.29 ± 0.44	0.94
20 ppm	1.82 ± 0.92	0.50	0.22 ± 0.12	0.52	5.4 ± 4.72^B^	0.04
30 ppm	2.45 ± 1.59	0.49	2.42 ± 3.72	0.67	3.45 ± 4.82	0.26
40 ppm	4.67 ± 4.55	0.89	2.48 ± 2.9	0.65	4.64 ± 3.26^b^	0.09
50 ppm	1.64 ± 1.99	0.54	0.75 ± 0.57	0.70	0.93 ± 0.55	0.92

ab;AB Different superscripts in the same column indicate statistically significant differences (*P* < 0.05 and *P* < 0.10 if upper or lowercase, respectively) based on the linear mixed model and Tukey corrected post hoc comparison of the type III LMM analysis of variance with Satterthwaite's method (n = 4).

### Crop 16S rRNA Gene Sequencing

**Figure 3:**
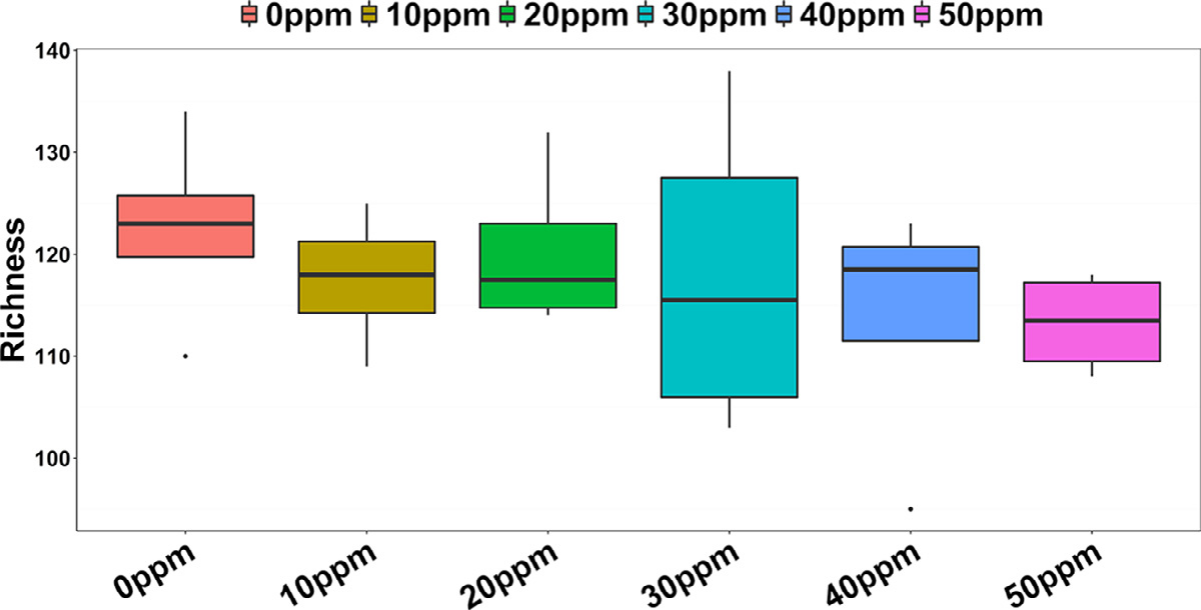
Microbial OTU alpha-diversity; richness calculated through the different levels of inclusion.

Both alpha- and beta-diversity of the crop microbial community, as measured through the richness index, Shannon index, and Bray-Curtis dissimilarity index and the Jaccard distance index, respectively, did not differ between PAA levels. Although especially richness appeared to decrease in a rather similar fashion in response to PAA level (Figure 3) when compared to the reduction in total bacterial concentration (qPCR), there were no linear (*P* = 0.78) or quadratic (*P* = 0.36) relationships observed.

The most abundant phylum across the treatments was Firmicutes (∼50%), followed by Proteobacteria (∼25%) and Cyanobacteria (∼20%), less dominant phyla was Actinobacteriota and Bacteroidota representing only ∼0.5% of the total population in all the samples (Table 5). Firmicutes decreased for all PAA levels compared to control (0 ppm), though most pronounced for 10 ppm PAA (*P* < 0.05) and 40 ppm PAA (*P* = 0.10). Similarly, albeit Cyanobacteria and Proteobacteria reads were smaller for all PAA levels compared to the control, this was significant for the 10 ppm group only (*P* < 0.01). The 10 most predominant genera (Table 6) were *Lactobacillus* accounting for almost 50% of the reads followed by Cyanobacteria (unassigned) and *Rickettsiales* (unassigned), each covering ∼20% of the total community, *Acinetobacter* (∼1.5%) and *Erwiniaceae* (unassigned), *Pseudomonas, Escherichia*-*Shigella, Ruminococcus* (*torques* group), unassigned Enterobacteriaceae and Enterobacterales all less than 1% of the total. *Lactobacillus* normalized reads were reduced for all PAA levels tested compared to the control (*P* < 0.05) apart from 50 ppm. *Flectobacillus* was also reduced for all PAA levels tested, though significantly so for the 20 ppm PAA group (*P* < 0.01). In contrast, while *Enterococcus* normalized reads were decreased for most PAA levels compared to the control (*P* < 0.05), they were increased for the 20 ppm PAA level (*P* < 0.05). Although the less dominant *Ruminococcus* (*torques* group) was found to be decreased for all PAA levels compared to the control (*P* < 0.05) apart from 40 ppm, *Subdoligranulum* reads were decreased for both 10 and 50 ppm PAA (*P* < 0.05), though noticeably increased at 40 ppm PAA (*P* < 0.05).

**Table 5:** Taxonomical composition at phylum level through the six different levels of inclusion of PAA administered through the study. The table depicts the calculated average of the relative abundance for each phylum at each treatment level ±SD calculated among the replicates.

	0 ppm	10 ppm	20 ppm	30 ppm	40 ppm	50 ppm
Firmicutes	57.7 ± 10.1A,a	58.9 ± 6.9^B^	47.1 ± 7.5	62.2 ± 7.7	51.7 ± 11.9^b^	55.6 ± 8.4
Cyanobacteria	18.4 ± 5.5	17.2 ± 6.7	21.7 ± 5.8	15.6 ± 5.1	19.3 ± 6.5	13.8 ± 3.9
Proteobacteria	23.5 ± 10.3^A^	23.5 ± 10.2^B^	31 ± 8.3	21.9 ± 3.1	28.7 ± 6.3	30.5 ± 5.2
Actinobacteriota	0.06 ± 0.06^A^	0.01 ± 0.03^B^	0.04 ± 0.05^B^	0.02 ± 0.02^B^	0.05 ± 0.07^B^	0.02 ± 0.03^B^
Bacteroidota	0.3 ± 0.2	0.3 ± 0.2	0.1 ± 0	0.2 ± 0.2	0.2 ± 0.1	0.1 ± 0.1
Unassigned	0.01 ± 0.01^A^	0 ± 0^B^	0.01 ± 0.02^B^	0.03 ± 0.02^B^	0 ± 0.01^B^	0.02 ± 0.01^B^

ab;AB Different superscripts in the same row indicate statistically significant differences (*P* < 0.05 and *P* < 0.10 if upper or lowercase, respectively) based on linear model analysis of the NEGBIN transformed reads (n = 4).

**Table 6:** Taxonomical composition at genus level through the six different levels of inclusion of PAA administered through the study. The table depicts the calculated average of the relative abundance for each genus amongst the 10 most abundant ones at each treatment level ±SD calculated among the replicates.

	0 ppm	10 ppm	20 ppm	30 ppm	40 ppm	50 ppm
Lactobacillus	57.42 ± 10.06^A^	58.88 ± 6.91^B^	46.23 ± 7.48^B^	62.07 ± 7.69^B^	51.41 ± 12.14^B^	55.5 ± 8.43
Cyanobacteria (Unassigned)	18.39 ± 5.46^a^	17.23 ± 6.73^b^	21.74 ± 5.83	15.63 ± 5.06	19.33 ± 6.46	13.76 ± 3.93
Rickettsiales (Unassigned)	20.77 ± 11.11	20.58 ± 9.6	27.74 ± 9.11	18.27 ± 2.68	25.88 ± 5.18	24.12 ± 8.23
Acinetobacter	1.61 ± 1.62	1.49 ± 1.26	1.15 ± 0.96	1.54 ± 1.22	0.84 ± 0.68	1.64 ± 1.25
Erwiniaceae (Unassigned)	0.25 ± 0.21^A^	0.11 ± 0.07^B^	0.37 ± 0.25	0.28 ± 0.19	0.44 ± 0.18	0.81 ± 0.87^B^
Pseudomonas	0.13 ± 0.09	0.15 ± 0.05	0.38 ± 0.31	0.36 ± 0.5	0.32 ± 0.18	0.25 ± 0.19
Escherichia-Shigella	0.09 ± 0.1	0.45 ± 0.27	0.64 ± 0.27	0.39 ± 0.42	0.17 ± 0.17	0.13 ± 0.1
Ruminococcus (torques group)	0.06 ± 0.07	0.02 ± 0.02^B^	0.07 ± 0.04^B^	0.04 ± 0.05^B^	0.06 ± 0.09	0.02 ± 0.02^B^
Enterobacteriaceae (Unassigned)	0.27 ± 0.19^A^	0.38 ± 0.16	0.33 ± 0.17	0.81 ± 0.77	0.5 ± 0.4	2.96 ± 2.68^B^
Enterobacterales (Unassigned)	0.14 ± 0.22^A^	0.13 ± 0.09	0.14 ± 0.06	0.08 ± 0.08	0.25 ± 0.09	0.33 ± 0.32^B^
Others	0.89 ± 0.47	0.59 ± 0.25	1.21 ± 0.67	0.53 ± 0.24	0.8 ± 0.32	0.49 ± 0.21

ab;AB Different superscripts in the same row indicate statistically significant differences (*P* < 0.05 and *P* < 0.10 if upper or lowercase, respectively) based on linear model analysis of the NEGBIN transformed reads (n = 4).

**Figure 4:**
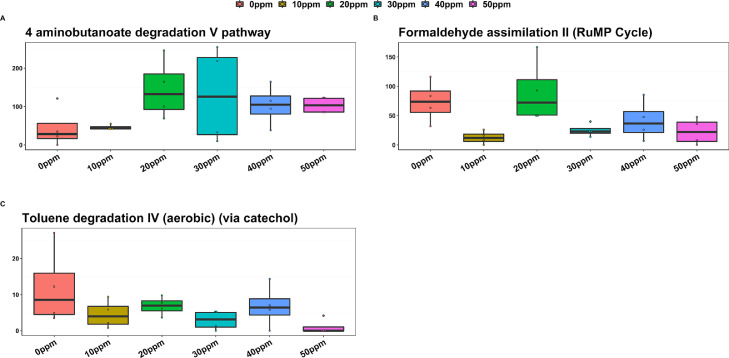
NEGBIN calculated differential abundance of TMM normalized predicted pathway abundance.

Figure 4 shows the NEGBIN calculated differential abundance of TMM normalized predicted pathway abundance for the different PAA levels tested. Diversity analysis did not reveal any statistically significant difference between the treatments apart from a tendency of 20 ppm PAA to an increased Shannon index (*P* = 0.05), pointing towards both qualitative and quantitative similarities of predicted metabolic pathways. The aminobutanoate degradation (V) pathway was noticeably less abundant in both control and for 10 ppm PAA compared to the rest of the treatments (*P* < 0.05), whereas albeit the pathway of formaldehyde assimilation II (RuMP Cycle) was less abundant in almost all the treatments, significant reduction was observed only for 10 ppm PAA compared to control (*P* < 0.01). Similarly, (aerobic) toluene degradation IV (via catechol) pathway was reduced in all treatments compared to control, although significant only for 50 ppm PAA (*P* < 0.01).

## DISCUSSION

Antimicrobial resistance poses a serious threat to animal and human health, mainly emphasized by reduced treatment effectiveness toward bacterial infections (World Health Organization, 2014). Finding alternative molecules to antimicrobials is among the strategies to decrease the AMR spread rate (Ghosh et al., 2019) through environment (Furtula et al., 2013) and livestock (Thanner et al., 2016). Here, we have assessed for the first time the potential antimicrobial alternative PAA for its effect on broiler performance, bacterial communities, and gut pH.

In particular, we tested 6 different levels of inclusion of PAA prepared fresh and administered daily from d 7 to d 14 of a 2-wk trial. The absence of observable side effects and mortality during the treatment week pointed toward the safety of this therapeutical approach, while we recorded an increased BW at d 14 in all treatment levels, though with less noticeable effects for the 10 and 50 ppm groups with some effects already visible at d 10. In general, average day14-BW through the 6 experimental conditions, was 466.67 g, thus 11.39% (60.3 g) lower than the target at day 14 (527 g) for the breed, whereas the average FI per bird throughout the experimental conditions between d 7 and d 14 was 367 g, which was 5.66% lower than FI/bird from d 7 to d 14, according to performance objectives for the breed (389 g).

Moreover, we found that incorporating the quadratic term in the mixed model was associated with a significant effect on both longitudinal BW and observations at d 14, indicating a possible dose-response effect. Therefore, the analysis of the 2 model components possibly pointed toward a range of optimum levels of inclusion for PAA in-water administration, with an optimal response at 20 ppm. This view was also supported by the observation that FI was most pronouncedly increased for 20, 30, and 40 ppm PAA.

The efficacy of the PAA treatment as a potential broad-spectrum antimicrobial alternative was confirmed by the observed reductions in crop bacterial concentration (Kitis, 2004), which reached a biologically relevant ∼1 Log_10_ reduction (Mayr et al., 2010) as PAA concentration increased, and without evident changes throughout the other gut locations analyzed.

While overall bacterial concentration was as per expectation throughout crop, jejunum, and ceca (Shang et al., 2018), these findings are consistent with the theoretical fast rate of PAA formation from its precursors, and its subsequent hydrolysis. Indeed, PAA reacts with water to form acetic acid and hydrogen peroxide (Zhao et al., 2007), thus it is assumable that without further encapsulating the precursors for further distal gut delivery (Chourasia and Jain, 2003), the likelihood of formation of active molecules is expected to be greater in the proximal gut. This concept represents the basis for further studies exploring alternative delivery methods of encapsulated PAA precursors to assess potential effect in the distal gut. Such proximal effect of PAA could also explain why colonic pH did not change, as PAA likely never reached the hind gut to modulate bacterial fermentation. However, it is worth to notice that colonic pH was found to be lower than the expected reported range of 7.0 to 8.0 (Ravindran, 2013; Skoufos et al., 2016). In addition, water acidification may improve performance, likely mediated through a reduction in pH of the gastrointestinal tract content (Hamid et al., 2018). The administration of PAA via precursors hydrolysis as presented here is not comparable with such approaches, as it does not cause variation of the gastrointestinal pH, which remains close to physiological levels.

Interestingly, it was noticed a monotonic inverse relationship between crop bacterial concentration and BW, with higher performance associated with lower bacterial load in the upper gut. Numerous studies have explored the potential relationship between especially the ileal and cecal microbiota and performance (Tiihonen et al., 2010; Torok et al., 2011; Ovi et al., 2021), whilst the number of studies that evaluate the role of the upper gut microbiota (Rinttilä and Apajalahti, 2013) or specifically of the crop microbiota (Ren et al., 2019) is poorly represented. For example, *Lactobacillus* is a primary symbiont in the crop (Yadav and Jha, 2019), whose decreased abundance has been correlated to a decreased intestinal activity of bile salt hydrolase produced by this genus, therefore, possibly leading to an increased host-lipid digestion and energy harvest (Chand et al., 2017), which in turn could potentially promote host lipid metabolism, energy harvesting and increased weight gain (Lin et al., 2013). This could likely explain the correlation between higher live weight and decreased *Lactobacillus* CSS normalized reads at increased PAA concentrations that were observed. On the other hand, *Lactobacillus* is traditionally recognized as a beneficial, probiotic strain for its action in the distal intestine, promoting performance, inhibiting pathogen growth by competition, and providing organic nutrients to the rest of the bacterial community (Ehrmann et al., 2002; Kabir, 2009; Pokorná et al., 2019; Sinha et al., 2020). Albeit *Lactobacillus* use as a probiotic has been validated by numerous studies focusing on the lower intestine, evidence provided here suggest that its reduction in the proximal gut could lead to better host-driven lipid digestion. Indeed, our findings not only indicate that microbiota modulation in the proximal tract is strictly correlated to performance amelioration but also suggest that the gastrointestinal section targeted should be taken into consideration when modulating specific genera whilst designing novel probiotic strategies.

We did not find any difference in crop microbial alpha- and beta-diversity due to treatment, which might indicate that the changes that we reported in terms of single phyla or genera did not affect the entropy of the general bacterial communities, whose variations where more quantitative than qualitative as indicated by the differences in bacterial concentration (qPCR) and marginally by the calculated OTU richness (16S sequencing).

We found that *Cyanobacteria* level was lower in treated birds than control, especially in the 10 ppm PAA group. The role of cyanobacteria within the gastrointestinal tract is not fully elucidated, although this phylum seems to be connected to a series of developmental and metabolic host functions (di Rienzi et al., 2013; Hu and Rzymski, 2022). Nevertheless, it cannot be ignored that at sequencing level, reads from this phylum could potentially also include chloroplast from indigested plant material (Willson et al., 2018).

We observed a drastic reduction of *Flectobacillus* for all PAA levels. This genus is known to contribute to mucosal immunity and homeostasis in other host species, especially IgM mediated by producing sphingolipids (Sepahi et al., 2016; Jin Song et al., 2019). This does not seem to accord with increased performance observed, although this apparent contrast could have arisen from its low relative abundance, supported by absence of effects on diversity parameters, and therefore, by its marginal role towards host interactions. It could also point towards a possible marginal sphingolipid role within the proximal gut, as opposite to the established positive roles known through the distal intestine (Vesper et al., 1999).

*Enterococcus faecium* has been described as a beneficial probiotic in Ross308 broilers, capable of improving performance whilst retaining carcass quality features (Gheisar et al., 2016), which could agree with our finding for *Enterococcus* genus CSS normalized reads increased in the 20 ppm PAA group. However, since its abundance for the other PAA levels remained lower than the levels observed in the control, such role at crop level might not be biologically relevant. Care should be taken when considering this strain as a probiotic in relation to broiler performance due to its associated vancomycin resistance (Cetinkaya et al., 2000; Ahmed and Baptiste, 2018). However, in our data, only a slight fold change increase was found for vanC in the 50 ppm PAA group, also associated to a high degree of variation.

Finally, we observed that the abundance of the 4-aminobutanoate degradation (V) predicted pathway was increased through all treatments ≥20 ppm, which has been correlated to increased insulin secretion in humans (Sanna et al., 2019) and could therefore be associated to an increased level of host glucose metabolism. From the 6 AMR genes analyzed, aadA, vanC, and mecA recorded sporadic higher relative abundance in some of the treatments, although the level of PAA at which this was observed was rather inconsistent. While it could be argued that, therefore, a reduction in AMR relative abundance triggered by PAA cannot be excluded, these observations were associated with relatively large degree of variation. Moreover, the absence of linearity between PAA level and AMR-DNA relative abundance may suggest these may be more likely chance findings rather than a systematic response to PAA intervention. However, the significance or tendency observed for the quadratic regression for tetQ and mecA, respectively, could point toward a selection pressure at some of the levels of inclusions and the need to optimize PAA administration concentration accordingly.

In conclusion, this first study strongly supports the view of the role of PAA as a possible broad-spectrum antimicrobial alternative, when administered in water for a week to young birds up to the age of 14 d. Our results also suggest that the modulation of the upper gut (i.e., crop) microbiota of young birds could contribute to changes in the host capability to metabolize specific nutrients, such as lipids and glucose, possibly leading to ameliorated performance in young birds. Therefore, our study not only suggests that the microbiota inhabiting the proximal intestine should be considered as a target for host-interaction modulation but also indicates that the established antimicrobial action of PAA could be applied in vivo to young chickens.
